# Amplicon Sequencing of Colorectal Cancer: Variant Calling in Frozen and Formalin-Fixed Samples

**DOI:** 10.1371/journal.pone.0127146

**Published:** 2015-05-26

**Authors:** Johannes Betge, Grainne Kerr, Thilo Miersch, Svenja Leible, Gerrit Erdmann, Christian L. Galata, Tianzuo Zhan, Timo Gaiser, Stefan Post, Matthias P. Ebert, Karoline Horisberger, Michael Boutros

**Affiliations:** 1 Division of Signaling and Functional Genomics, German Cancer Research Center (DKFZ) and Department of Cell and Molecular Biology, Medical Faculty Mannheim, Heidelberg University, Heidelberg, Germany; 2 Department of Medicine II, University Hospital Mannheim, Medical Faculty Mannheim, Heidelberg University, Mannheim, Germany; 3 Department of Surgery, University Hospital Mannheim, Medical Faculty Mannheim, Heidelberg University, Mannheim, Germany; 4 Institue of Pathology, University Hospital Mannheim, Medical Faculty Mannheim, Heidelberg University, Mannheim, Germany; Seoul National University College of Medicine, REPUBLIC OF KOREA

## Abstract

Next generation sequencing (NGS) is an emerging technology becoming relevant for genotyping of clinical samples. Here, we assessed the stability of amplicon sequencing from formalin-fixed paraffin-embedded (FFPE) and paired frozen samples from colorectal cancer metastases with different analysis pipelines. 212 amplicon regions in 48 cancer related genes were sequenced with Illumina MiSeq using DNA isolated from resection specimens from 17 patients with colorectal cancer liver metastases. From ten of these patients, paired fresh frozen and routinely processed FFPE tissue was available for comparative study. Sample quality of FFPE tissues was determined by the amount of amplifiable DNA using qPCR, sequencing libraries were evaluated using Bioanalyzer. Three bioinformatic pipelines were compared for analysis of amplicon sequencing data. Selected hot spot mutations were reviewed using Sanger sequencing. In the sequenced samples from 16 patients, 29 non-synonymous coding mutations were identified in eleven genes. Most frequent were mutations in TP53 (10), APC (7), PIK3CA (3) and KRAS (2). A high concordance of FFPE and paired frozen tissue samples was observed in ten matched samples, revealing 21 identical mutation calls and only two mutations differing. Comparison of these results with two other commonly used variant calling tools, however, showed high discrepancies. Hence, amplicon sequencing can potentially be used to identify hot spot mutations in colorectal cancer metastases in frozen and FFPE tissue. However, remarkable differences exist among results of different variant calling tools, which are not only related to DNA sample quality. Our study highlights the need for standardization and benchmarking of variant calling pipelines, which will be required for translational and clinical applications.

## Introduction

Due to recent advances in deep sequencing technologies, remarkable insights have been gained on the alterations acquired by colorectal cancer (CRC) genomes during the carcinogenic process, largely expanding our view on CRC genomic progression [1–3]. The promise that after structural characterization of cancer genomes, clinical decision-making would be guided by individual genomic tumor profiles, however, remains to be fulfilled. Nevertheless, the development of novel targeted therapeutics highlights the need for reliable and cost effective methods for molecular characterization of cancer genomes to identify patients that ultimately respond to treatment on the basis of druggable mutations, predictive alterations or acquired resistance markers.

Targeted sequencing based on PCR amplicons represents a feasible approach for evaluation of actionable mutations, mutational hot spots or predictive alterations in cancer genomes for clinical studies. Compared to genome-wide or exome-wide sequencing, a high depth of sequencing (>1000 reads) at the genomic loci of interest can be reached, thus facilitating detection of low-frequency variants in heterogeneous tumor samples admixed with stromal cells [4,5]. Moreover, due to the comparably low number of base pairs to be sequenced per patient, multiple samples, also for longitudinal analysis, can be analyzed in parallel on bench-top machines such as Illumina MiSeq, lowering costs and potentially allowing routine clinical application in the near future.

However, for clinical application and for translational studies on archived clinical samples, many problems remain to be solved. Most widely available specimens for clinical diagnostics and biomarker studies are formalin-fixed, paraffin-embedded (FFPE) tissues from pathology archives, as their long-term storage is relatively simple and cost efficient compared to frozen material. However, it is known that formalin fixation leads to covalent linking of DNA, RNA and protein by methylene bridges, deamination and oxidation reactions, formation of cyclic base derivatives and also to DNA fragmentation [6]. These DNA alterations hamper sequencing technologies leading to less robust results and difficulties in interpreting data from sequencing experiments. Furthermore, a gold standard method for analysis of next-generation sequencing (NGS) data is lacking and quality assurance programs are not launched yet. Different bioinformatic analysis tools and pipelines have been developed for NGS data. However, it appears that reproducibility between them needs to be improved [7]. Moreover, statistical models for variant discovery and variant evaluation, designed for whole-exome or whole-genome data consisting of many samples with low coverage, may not be optimal for small amplicon datasets with few targeted regions. Thus, there is no generally accepted standard on how to perform variant calling on amplicon sequencing data. These problems highlight the need for sample preparation and data analysis pipelines optimized for amplicon sequencing of clinical samples.

In this study, we describe an experimental and bioinformatic pipeline for amplicon sequencing of clinical fresh frozen and FFPE samples from CRC. Special focus is drawn on preparation of sequencing libraries from low-quality FFPE samples. The bioinformatics pipeline, using an adapted Genome Analysis Toolkit (GATK) Unified Genotyper, is explained in detail and compared with other commonly used variant calling methods with respect to their suitability for amplicon sequencing using FFPE material.

## Materials and Methods

### Patients

Thirty-three samples from 17 patients who underwent resection of liver metastasis of CRC in the Department of Surgery, University Hospital Mannheim, between February 2012 and February 2013 were included in this study. For all of these patients, either fresh frozen or formalin-fixed paraffin-embedded (FFPE) tissue was used for DNA isolation. From 10 patients, paired frozen and FFPE tissue was available for study and from 5 patients, matched primary tumors could be obtained from the archives of the Institute of Pathology, University Hospital Mannheim. Additionally, one matched primary-metastasis pair from a neuroendocrine carcinoma of the small bowel (Pat05), primary culture material from one patient (Pat16), material from a prostate cancer patient and cell lines DLD-1, HCT116, HT55, HUH7, HEK293T, HS68 and SW480 were included in sequencing runs and analysis for other projects or as controls. Samples were analyzed in two sequencing runs, one patient (Pat13) was analyzed in both runs as control. All cell lines were obtained from ATCC. Information about patients can be found in S1 Table.

### Ethics approval

Ethics board approval was obtained from the Medical Ethics Commission II of the Medical Faculty Mannheim, Heidelberg University, Mannheim, Germany (No. 2012-293N-MA, 2013-841R-MA, 2014-551N-MA). Written informed consent from the donors of tissue samples was obtained for the use in research.

### Sample preparation

#### Frozen samples and cell lines

**Fig 1:**
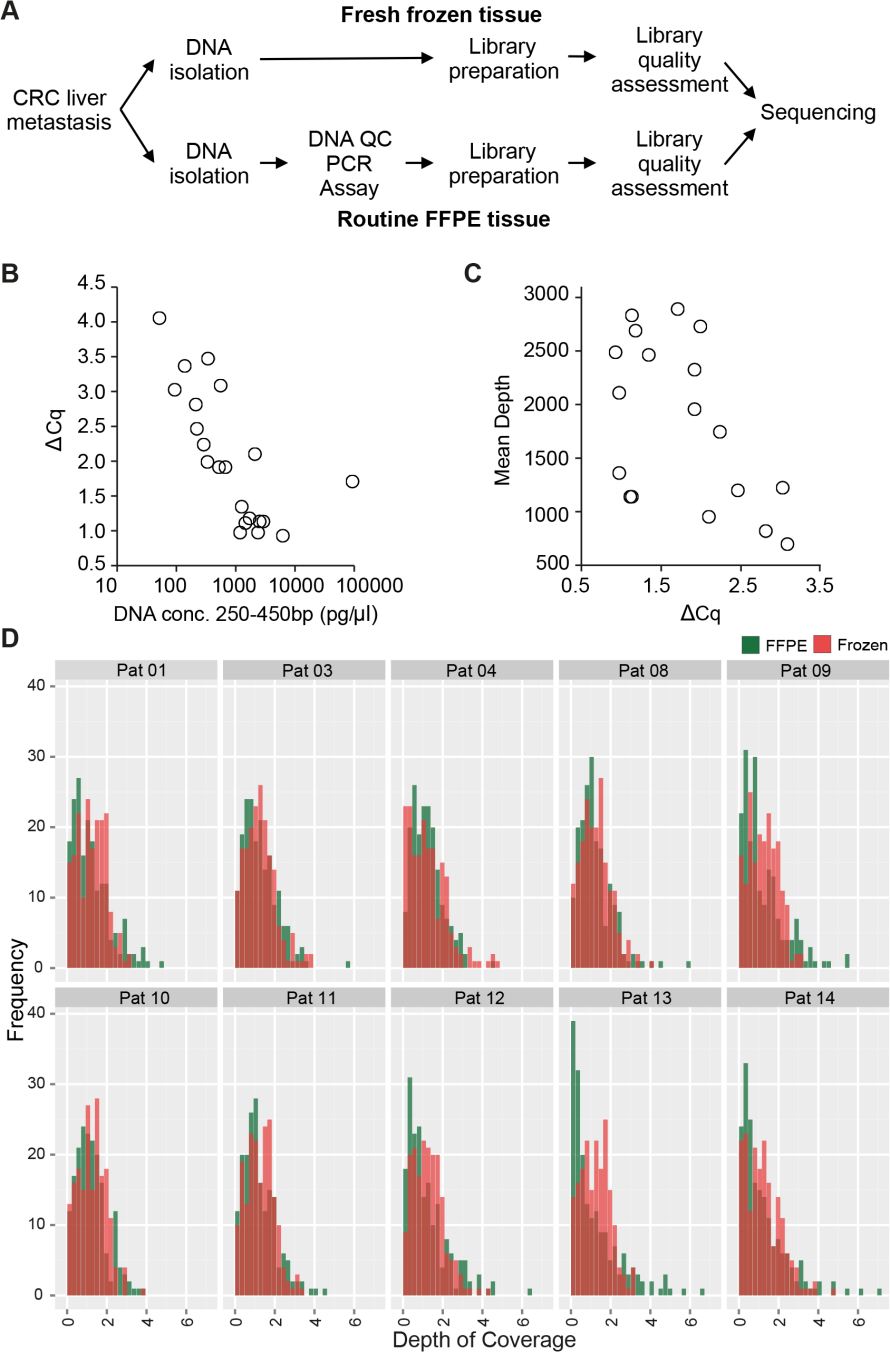
Depth of Sequencing correlates with DNA quality. (A) Sample preparation workflow. DNA was isolated from fresh frozen or FFPE CRC liver metastasis resection specimens with Qiagen Blood and Tissue or FFPE kit, respectively. Frozen samples then directly underwent sequencing library preparation, pooling of libraries, quality control and sequencing. FFPE samples were additionally tested for DNA quality by qPCR. Library quality was tested with Bioanalyzer. For samples with low amounts of correctly sized DNA amplicons (fragments at 310bp), new libraries were prepared with higher starting DNA concentrations and re-analyzed with Bioanalyzer. Samples with yet low amounts of DNA with correct size and highly fragmented DNA were excluded. (B) ΔCq-values of quality control PCR indicate poor sample quality. DNA concentration of fragments between 250bp and 450bp after library preparation was calculated with Agilent Bioanalyzer and plotted against ΔCq values of FFPE quality control PCR. (C) higher ΔCq-values correlate with lower mean depth of sequencing. (D) Coverage distribution of amplicons from all paired FFPE and frozen samples, normalized to total sample coverage. Frozen samples had a mean depth of 4,622, FFPE samples 1,852.

Samples from hepatic metastases from CRC patients were transported in RPMI cell culture medium and were snap frozen on dry ice and subsequently stored at -80°C. DNA isolation was done with the Qiagen DNeasy Blood & Tissue Kit (Qiagen, Hilden, Germany) according to the manufacturers recommendations, including RNAse digestion (Fig 1A). Cell lines were pelleted and DNA was isolated with the same protocol. Extracted DNA was diluted and directly used for preparation of sequencing libraries.

#### FFPE samples

Tissue from hepatic metastases had been fixed in formalin und embedded in paraffin during routine pathological work-up. Suitable blocks were chosen and five 10μm slices were used for DNA extraction without microdissection. A slide stained with haematoxylin and eosin (H&E) from each block was used to estimate the tumor cell content of the corresponding slices by two investigators (TG and JB) using a double-headed microscope. DNA was isolated using the Qiagen QIAamp DNA FFPE Kit according to the manufacturer’s instructions. DNA was eluted in 40μl Buffer ATE and concentrations were measured with NanoDrop 2000 (NanoDrop, Wilmington, USA) and Qubit BR kit (Life Technologies, Darmstadt, Germany). Isolation yielded between 4.8μg and 22.8μg (mean 10.23μg) when measured with the Qubit BR kit. Detailed information about preparation of FFPE samples can be found in S2 Table.

### Library Preparation

DNA quality of FFPE samples was evaluated by determining the amount of amplifiable DNA using the FFPE QC PCR (Illumina, San Diego, USA) according to the manufacturer’s recommendations. Mean **Δ**Cq-value of all FFPE samples was 2.0 (Median 1.9, Min 0.9, Max 4.1). Nine samples (47%) had a **Δ**Cq value higher than the recommended 2.0 (S2 Table). TruSeq Amplicon Cancer Panel (Cat. No. FC-130-1008, Illumina) libraries were prepared with recommended DNA amounts (150ng for fresh frozen material and cell lines, 250ng for FFPE samples). The panel includes 212 amplicons of 170–190bp lenght, targeting mutational hot spots in 48 cancer related genes. Amplicon regions are depicted in S3 Table.

Bioanalyzer (Agilent Technologies, Böblingen, Germany) was used to confirm successful library amplification and library quality of FFPE samples by assessing concentration of DNA with aspired size (~310bp) and short DNA fragments (<150bp). To compare amounts of DNA within the desired size region, the concentration of DNA amplicons in the range of 250–450bp was calculated. Concentration of DNA with a size between 250bp and 450bp varied greatly between 51.7 and 93831.9 pg/μl (mean 5675.1 pg/μl, median 672.2 pg/μl) within the libraries of different samples and inversely correlated with **Δ**Cq values (Spearman’s Coefficient: -0.805, Fig 1B, S2 Table). For the samples with low DNA concentrations at the 310bp amplicon, library preparation was repeated using highest possible DNA amounts (S1 Fig, S2 Table). Bioanalyzer revealed higher concentrations of DNA around 250–450bp (365.3 pg/μl—5669.8 pg/μl; mean 6190.9 pg/μl; median 1996.3 pg/μl), however, with significant background of short DNA fragments. After PCR clean-up of libraries, short DNA fragments were reduced, but three samples also showed diminished amounts of the 310bp amplicon and were thus excluded from sequencing.

### Data processing

**Fig 2:**
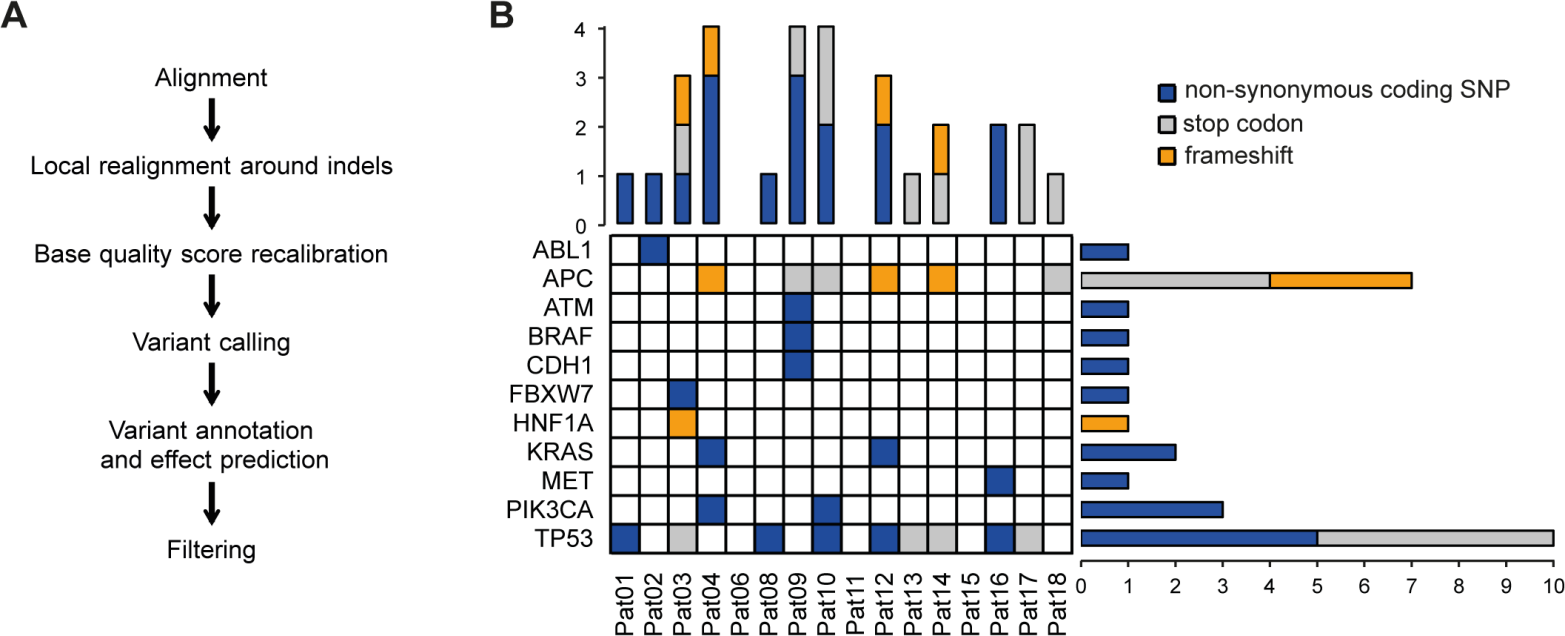
Amplicon Sequencing identifies hot-spot mutations in CRC metastases. (A) Sequencing analysis workflow. Sequence alignment files underwent local-realignment around Indels, left alignment and base quality score recalibration. After variant calling with GATK Unified Genotyper, annotation and effect prediction of detected variants was done using SnpEff. Raw variants of all samples were filtered by custom parameters with SnpSift. Variants included in the 1000 Genomes Project data were excluded to only obtain somatic mutations in cancer. (B) High frequency of TP53 and APC mutations among somatic mutations identified in CRC liver metastases (frozen and FFPE tissue). Colored fields represent presence of a nonsynonymous coding SNP (blue), a mutation leading to a stop-codon (grey) or a frameshift mutation (orange). Bars sum up mutations present in each patient (vertical bars) or each mutated gene (horizontal bars). Of note, some genes contain more than one mutation.

Bioinformatic analysis pipeline is shown in Fig 2A. Reads were aligned against hg19 reference genome using the BWA algorithm implemented the MiSeq software (MiSeq Reporter v2.2.29). BAM files were quality-checked with FASTQC (v.0.9.5; http://www.bioinformatics.babraham.ac.uk/projects/fastqc/). Indels in sequence alignment files were left-aligned and local realignment around Indels was done with the RealignerTargetCreator and the IndelRealigner tools from the Genome Analysis Toolkit (GATK, version 2.4–9) [8]. Base quality score recalibration was performed. Duplicate mapping and marking was not deemed suitable for amplicon sequencing and thus omitted.

### Unified Genotyper pipeline

#### Variant calling

Unified Genotyper from the GATK (version 2.4–9) was used for variant calling. All samples were processed in parallel and split into individual variant files for each sample after variant calling. Maximum coverage per locus was increased from the default 250 to 9,000,000 to take into account the high depth of amplicon sequencing. (Downsampling to lower depth is done in whole-exome studies to increase speed by saving memory). The minimum confidence threshold for calling was set to 10, the minimum confidence threshold for emitting to 30. SNPs and Indels were evaluated simultaneously. A region list of all amplicons was used to define regions for single nucleotide polymorphism (SNP) and Indel calling to increase analysis speed. As an alternative, the Unified Genotyper pipeline was used by processing each sample individually, otherwise the same parameters were used.

#### Variant annotation and effect prediction

SnpEff (version 2.0.5, http://snpeff.sourceforge.net/) [9] was used for variant annotation and effect prediction and the GATK VariantAnnotator tool was run with the—A SnpEff option to add the SnpEff annotations with the highest biological significance for each variant to the variant calling format (vcf) files. Subsequently, the vcf file with information about all sequenced samples was split into individual sample variant files using the GATK SelectVariants program. Variants were annotated with the variant frequencies in the 1000 genomes project using the SnpSift (http://snpeff.sourceforge.net/SnpSift.html) annotate feature [9].

#### Variant filtering

SnpSift from the SnpEff package was used for filtering of raw variants. The following quality-filter criteria were applied: quality by depth greater than 0.8 (QD > 0.8), total depth for calling variants at a specific locus greater than 200 (DP > 200), Fisher strand (Phred-scaled p-value using Fisher's Exact Test to detect strand bias) smaller than 70 (FS < 70), minimum variant confidence greater than 1500 (QUAL > 1500), mapping quality greater than 40 (MQ > 40) and mapping quality rank sum test higher than -15 (! exists MQRankSum | MQRankSum > -15). Filter criteria had been optimized by explorative analysis. Moreover, only the coding variants were selected with the following expressions: (SNPEFF_EFFECT = 'NON_SYNONYMOUS_CODING') | (SNPEFF_EFFECT = 'CODON_CHANGE_PLUS_CODON_DELETION') | (SNPEFF_EFFECT = 'CODON_DELETION') | (SNPEFF_EFFECT = 'FRAME_SHIFT') | (SNPEFF_EFFECT = 'STOP_GAINED')). All variants present in the 1000 Genomes data were excluded to obtain only somatic mutation data and exclude common germline variants. Variant recalibration was not done due to the nature of targeted sequencing data and the relatively small dataset.

### SAMtools mpileup/BCF-tools pipeline

SAMtools (version 0.1.18) mpileup was used to generate raw variant calls with the—u (generate uncompress BCF output),—f (faidx indexed reference sequence file),-D (output per-sample DP),-S (output per-sample strand bias P-value) options and hg19 as reference genome, processing all samples in parallel. Maximum per-sample depth for Indel and SNP calling was set to 10,000. Bcftools view with—bvcg options (output BCF file format, output potential variant sites only, call SNPs, call genotypes at variant sites) was used for variant calling. Data were processed and variants were annotated as for GATK data described above. Variants at loci with a depth of less than 50 were filtered out, as well as all non-coding variants and all variants present in the 1000G data.

### Illumina Somatic Variant Caller pipeline

MiSeq on-board software Somatic Variant Caller was run with default parameters. Vcf files containing variant information were downloaded from Basespace. Subsequently, they were annotated with 1000G variant frequencies. All non-coding, silent, synonymous and unknown variants were filtered out, as well as all variants present in 1000G data. Moreover, all variants at a locus with coverage of < 200, variants with a variant frequency < 0.05 or with a genotype quality less than 100 were excluded.

### Data analysis and visualization

Filtered variants were exported from variant files into tab-delimited files using SnpSift and concatenated into a single tab-delimited file including all variants of all patients. Descriptive statistics and data visualization was performed using Microsoft Excel and R packages (http://www.r-project.org/). Venn diagrams were made using venny (http://bioinfogp.cnb.csic.es/tools/venny/index.html) and jvenn [10]. The Integrative Genomics Viewer was used for analysis and visualization of specific mutated loci [11].

The amplicon sequenceing data of all samples were deposited in the European Nucleotide Archive (ENA) and can be accessed with accession number PRJEB8754.

### Sanger sequencing

Sanger sequencing was performed to evaluate KRAS exon 2 and BRAF exon 15 statuses as described here [12]. Briefly, genomic DNA was extracted from FFPE tumor tissue after manual macro-dissection using the QIAamp DNA Micro kit (Qiagen, Hilden, Germany). The following PCR primers were used for amplification: 5-AACACATTTCAAGCCCCAAA-3′ (BRAF-F), 5′-GAAACTGGTTTCAAAATATTCGTT-3′ (BRAF-R), 5′-AGGCCTGCTGAAAATGACTGAATA-3′ (KRAS-F), 5′-CTGTATCAAAGAATGGTCCTGCAC-3′ (KRAS-R), 5′-

Thermal cycling conditions were 5 min at 94°C, followed by 35 cycles of 94°C for 30 seconds, 53°C (BRAF) or 60°C (KRAS) for 30 seconds and 72°C for 30 seconds followed by a final incubation at 72°C for 7 minutes. After dye-terminator sequencing using the PCR amplification primers, analyses by capillary electrophoresis were performed on a 3130 Genetic Analyzer (Applied Biosystems, Foster City, CA).

## Results

### Depth of sequencing correlates with DNA quality

We sequenced 212 amplicon regions in 48 cancer related genes with Illumina MiSeq using DNA isolated from resection specimens from 17 patients with CRC liver metastases. From ten of these patients, paired fresh frozen and routinely processed FFPE tissue was available for comparative study. Sequencing statistics and DNA quality measurements were analyzed to evaluate differences of FFPE and frozen material (Fig 1A).

The number of paired reads and paired reads mapped was significantly higher in frozen samples compared with FFPE samples, however the percentage of mapped/raw reads was only 78% compared with 96% in FFPE (Table 1). Mean sequencing quality (Phred score 38 vs. 37) was gradually higher in FFPE samples compared to frozen samples; also the GC content was higher in FFPE than in frozen tissue (49% vs. 45%). Detailed sequencing statistics for each frozen and FFPE sample are shown in S4 Table. Frozen samples had a mean depth of 4,622 reads, FFPE samples of 1,852 reads. In FFPE samples, we investigated the correlation of sequencing depth with DNA quality measured by quality control PCR. This step is performed before library preparation and estimates the amount of amplifiable DNA as a surrogate for functional DNA quality (Fig 1B and 1C). We found that higher **Δ**Cq-values, indicative of lower DNA quality, correlated with lower mean depth of sequencing (Pearson Coefficient -0.505, Fig 1C). Of note, higher **Δ**Cq-values also correlated with higher GC-content of the samples (Pearson Coefficient 0.488, S2 Fig) while the depth of sequencing appeared to be independent of mean GC-content of the sequenced sample (S2 Fig). Fig 1D shows histograms of the coverage of amplicons for each paired FFPE and frozen samples, normalized to total coverage of the sample. FFPE samples tend to have a less balanced distribution of coverage on the different amplicons than frozen samples.

**Table 1:** Sequencing statistics of all patient samples.

	FFPE	Frozen
# Raw Paired Reads	423,355	1,278,334
# Paired Reads Mapped	407,493	984,164
% Mapped/Raw	96%	78%
Mean Depth	1,852	4,622
%GC	49	45
Mean Seq. Quality (Phred score)	38	37
Mean Mapping Quality	49.63	48.98

These data indicate that sequencing performance correlates with DNA quality of sequenced FFPE samples.

### High concordance of mutations identified in frozen and FFPE samples from CRC metastases

Recent large scale projects have identified the most common mutations occurring in CRC [1]. Sequencing 212 amplicon regions in 48 cancer related genes, we analyzed variant calls using an adapted Unified Genotyper analysis pipeline.

In the sequenced tumor samples from 16 patients (frozen and/or FFPE), a total of 29 mutations were identified in eleven genes after excluding all non-coding mutations, all synonymous variants, and all non-harmful variants present in the 1000 genomes data (Fig 2A–2B). The number of mutations per patient varied from zero to four, mean number of mutations per patient was 1.8. Of the mutations, 16 were SNPs, four were Indels leading to a frameshift and nine to a stop codon. The most frequently mutated gene was TP53, which showed 10 mutations in nine of the patients. We observed seven APC mutations in six patients, while KRAS and PIK3CA were mutated two and tree times, respectively (Fig 2B).

**Fig 3:**
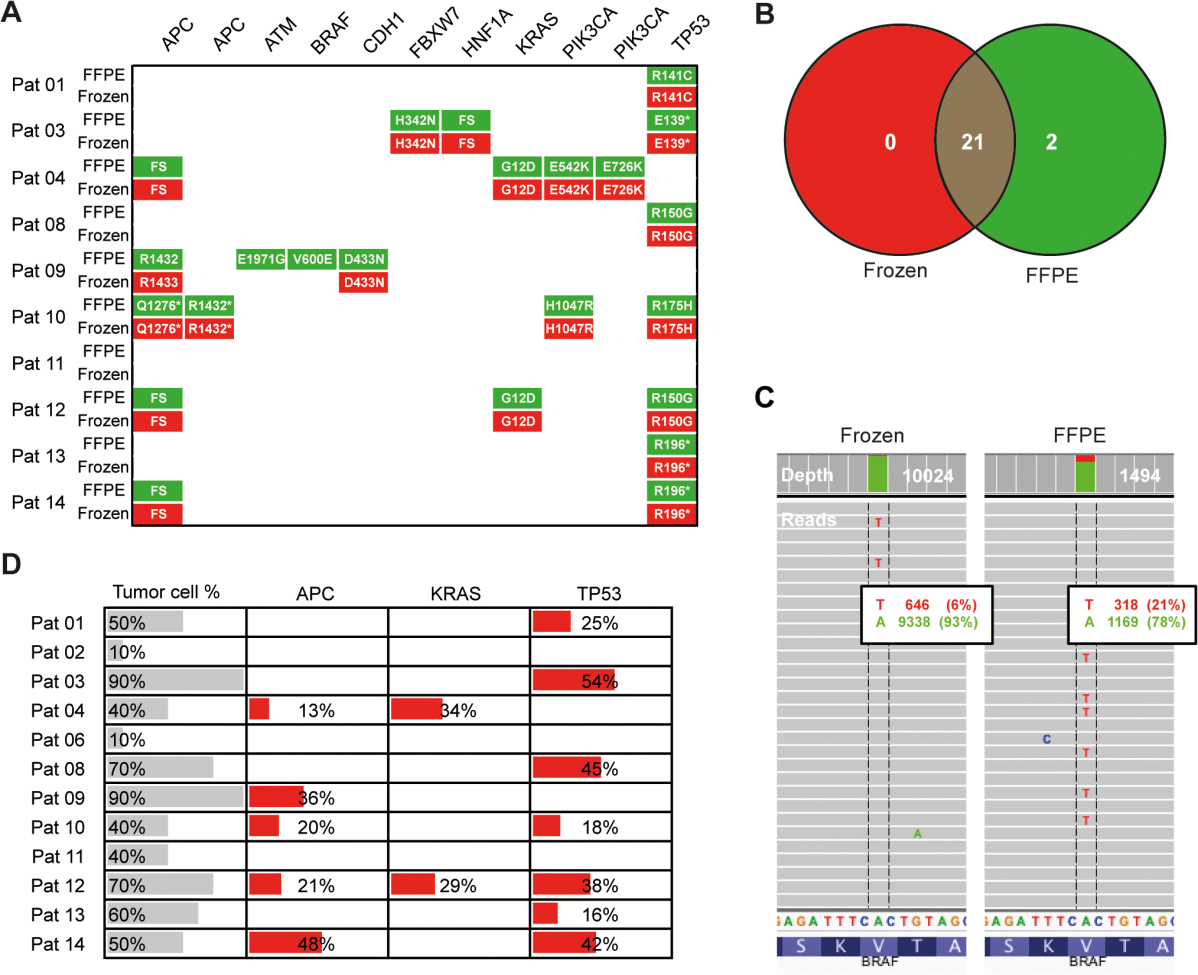
Paired frozen and FFPE samples of CRC liver metastases have a high concordance of mutations in hotspot cancer genes. (A) GATK Unified Genotyper variant calling pipeline was used to identify non-synonymous coding mutations in FFPE (green) and frozen samples (red). (B) Venn-Diagram of non-synonymous coding mutations identified in FFPE and frozen samples. (C) Representative images of reads mapped to the site of BRAF V600E mutation identified in FFPE but not in frozen tissue of patient 09, displayed with the Integrative Genomics Viewer. (D) Variant frequency of selected mutations and estimated tumor cell content analyzing FFPE samples.

DNA from FFPE tissues may have alterations due to the process of fixation in formalin. We compared the variants identified in paired frozen and FFPE tissues. In ten sequenced patients with paired frozen and FFPE tissue, 23 mutations were identified in FFPE samples and 21 mutations in frozen samples, thus a concordance of 91% could be observed (Fig 3A and 3B). The two non-matching mutations (BRAF V600E and ATM E1971G) were both identified in the FFPE but not in the frozen sample of patient 09. Sanger sequencing of the BRAF mutational hotspot in exon 15 was performed, revealing V600E mutation. Of note, six percent of >10,000 reads at the BRAF V600E locus in the frozen sample showed the alternative base “T”, which however did not lead to a variant call with Unified Genotyper pipeline (Fig 3C).

The correlation between observed percentage of tumor cells on representative FFPE slides and calculated variant frequency for selected mutations was moderate (Fig 3D).

These data show that sequencing of FFPE tissue can lead to overall similar results as sequencing frozen material and could thus be a feasible approach for routine clinical samples.

### Low reproducibility of variant calling in FFPE and frozen tissue with different bioinformatics pipelines

**Fig 4:**
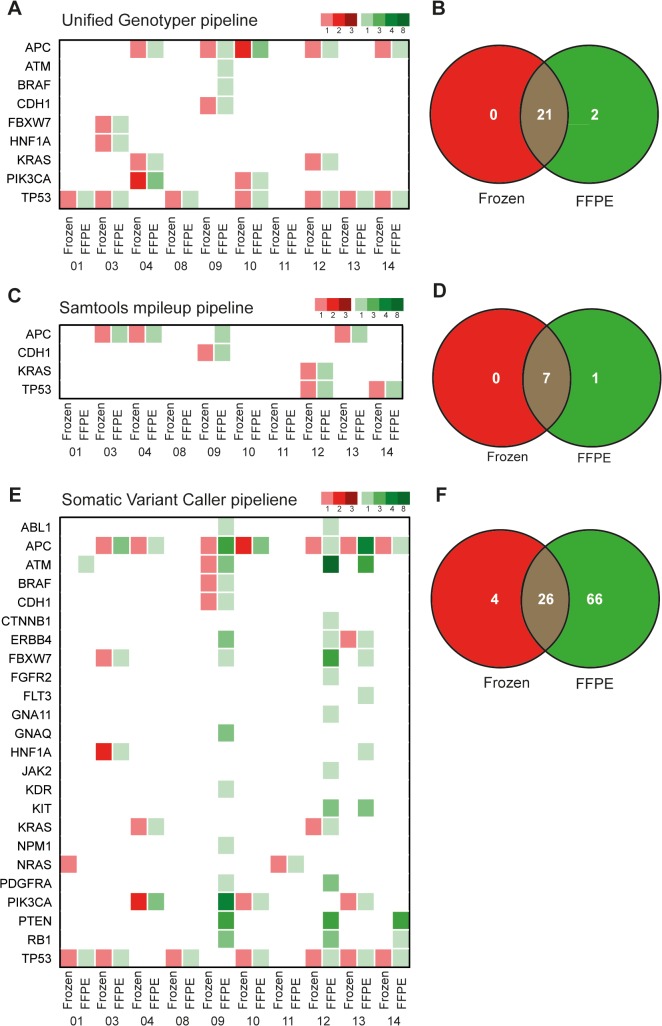
Comparison of different methods for variant calling. Mutations identified in matched frozen and FFPE tissue of CRC liver metastases detected with (A, B) Genome Analysis Toolkit (GATK) Unified Genotyper (C, D) Samtools mpileup/Bcftools and (E, F) Somatic variant caller. Green color represents FFPE samples, red represents frozen, color intensities represent number of non-synonymous coding mutations per gene.

Low reproducibility between different variant calling pipelines has been reported for whole-genome or whole-exome sequencing data [7]. To test whether this problem also occurs with amplicon sequencing data, we compared different tools for variant calling in order to test reproducibility of our results. We observed marked differences between different variant calling software (Fig 4). Compared to Unified Genotyper pipeline (Fig 4A and 4B) Samtools/BCFtools found five of the mutations identified with the Unified Genotyper pipeline (patient 04 APC, patient 09 CDH1, patient 12 KRAS and TP53 and patient 14 TP53). The APC mutation of patient 09 was also identified at the same locus but only in the frozen sample. However, two additional APC frameshift mutations in patients 03 and 13 were called only by Samtools/BCFtools. In contrast, 15 mutations called with the Unifed Genotyper pipeline in both FFPE and frozen as well as two mutations called only in FFPE tissue were not identified with Samtools/BCFtools. Thus, Samtools/BCFtools as used in our pipeline seems to be less sensitive, although it may identify additional small Indels leading to frameshift mutations (Fig 4C and 4D). Moreover, results from Illumina MiSeq on-board Somatic Variant Caller pipeline are shown in Fig 4E and 4F. Notably, this pipeline appears to call variants in both frozen and FFPE samples that are not identified by other pipelines.

Regarding the paired primary CRCs we analyzed from patients 04, 10, 11 and 14, Illumina Somatic Variant Caller again called more variants than others, especially in patient 04 (S5 Table). Cell lines that were included as controls are shown in S6 Table. In the cell lines, almost identical results were obtained with the Unified Genotyper pipeline and Illumina Somatic Variant Caller, while Samtools mpileup/Bcftools was less sensitive.

All variant data from patients and cell lines obtained with different variant calling pipelines can be found in S7 Table.

These data indicate that remarkable differences exist among results of different variant calling pipelines, which are not only related to DNA sample quality.

### Sensitivity and specifity of amplicon sequencing with respect to different variant calling pipelines using frozen and FFPE tissues

To evaluate sensitivity and specifity of amplicon sequencing analyzed with different bioinformatics tools, we performed Sanger sequencing of KRAS exon 2. As shown in Table 2, sensitivity and specifity were 100% using Unified Genotyper with DNA isolated from frozen samples. In FFPE samples, one discordant case (patient 02) was noted, which had KRAS c.38G>A mutation according to Sanger sequencing. However, of note, Sanger sequencing was performed with material from the primary tumor and the metastatic piece analyzed with amplicon sequencing had estimated tumor content of only 10%. In addition, none of the reads had the mutated variant at the mutation locus (S3 Fig). Frozen tumor sample was not available from this patient. Regarding other variant calling pipelines, Samtools/BCFtools failed to identify KRAS mutation of patient 04, while Somatic Variant Caller had a false positive call in patient 02 FFPE sample, missing the mutation at codon 38.

**Table 2:** KRAS mutations identified by sanger sequencing compared to deep amplicon sequencing analyzed with different variant calling tools.

No.	Sanger	UG frozen	UG FFPE	SAM frozen	SAM FFPE	SVC frozen	SVC FFPE
Pat02	c.38G>A	NA	0	NA	0	NA	c.423T>A
Pat03	0	0	0	0	0	0	0
Pat04	c.35G>A	c.35G>A	c.35G>A	0	0	c.35G>A	c.35G>A
Pat06	0	NA	0	NA	0	NA	0
Pat08	0	0	0	0	0	0	0
Pat09	0	0	0	0	0	0	0
Pat11	0	0	0	0	0	0	0
Pat12	c.35G>T	c.35G>T	c.35G>T	c.35G>T	c.35G>T	c.35G>T	c.35G>T
Pat17	0	0	NA	0	NA	0	NA
Pat18	0	0	NA	0	NA	0	NA

UG, Unified Genotyper pipeline; SAM, Samtools mpileup/Bcftools pipeline, SVC, Somatic Variant Caller; NA, not available

Additionally, human cancer cell lines were analyzed to test concordance of variant calling pipelines irrespective of sample quality and to evaluate suitability of filter criteria. As shown in S4 Fig, a high concordance is observed between variant loci identified in cancer cell lines after filtering poor quality and non-harmful variants. Moreover, almost all of the variant loci in cell lines HCT116, HT55, HUH7 and SW480 identified with Unified Genotyper pipeline were also identified by large scale databases Cell Line Encyclopedia [13] and COSMIC [14], while discordant loci were largely eliminated from our data upon filtering (S4 Fig).

Accordingly, in CRC metastases substantial differences can be observed between raw datasets and datasets after filtering variants by quality measures and functional annotations. Variant count is substantially reduced, while concordance between frozen and FFPE, as well as between different variant calling pipelines increases. Results are presented in S5 Fig.

### Processing all sequence alignment files together for variant calling is more sensitive than separately

Processing many samples together for variant calling is generally recommended for whole-genome or whole-exome sequencing data in order to increase the number of reads at specific loci. However, it is not known whether this is also beneficial for deep amplicon sequencing, since it might lower the impact of rare variants only present in a subset of tumor cells in few samples. In contrast, it might increase sensitivity for common mutations present in many samples. We observed a general increase in sensitivity for variant calling when samples were processed in parallel (S6A Fig and S6B Fig) compared to separate processing with otherwise identical pipeline and filter criteria (S6C Fig and S6D Fig). Separate variant calling identified no additional mutation compared to combined variant calling, but missed three mutations in frozen samples and five mutations in FFPE samples. Hence, even in high-depth amplicon sequencing data, processing samples in parallel appears to be beneficial.

## Discussion

We performed amplicon sequencing of hot-spot mutational regions in cancer related genes in clinical samples from 16 patients with metastatic CRC with Illumina MiSeq. From ten patients, we compared results of fresh frozen and FFPE tissue and observed a high concordance of variant calls using GATK Unified Genotyper pipeline with adapted filter criteria and processing variant calling on all samples in parallel. Thereby, we illustrate the general feasibility of amplicon sequencing in FFPE tissue. However, we observed marked differences among tested variant calling pipelines even in this small dataset, highlighting the importance of benchmarking and development of more robust variant calling methods. Moreover, preparation of sequencing libraries with DNA from low quality FFPE samples remains challenging. Here, we prepared sequencing libraries also for samples with poor quality by increasing input DNA and demonstrated successful library amplification by analysis with Agilent Bioanalyzer. However, we observed that samples with poor DNA quality also had lower sequencing coverage and were more problematic for variant calling.

Observed mutation frequencies were in line with literature data for APC, TP53, PIK3CA and KRAS being most frequently altered in CRC [1]. TP53 but not APC had the highest mutation frequency in our cohort, which may be due to the fact that we sequenced metastases and TP53 mutation is known to be a comparably late step in the carcinogenic process [15]. APC mutations occurred in lower frequency than expected. It is likely that mutations in regions not targeted by our approach have been missed.

Mutation calling in NGS data is challenging due to various potential sources of error, including not only sequencing errors, but also artifacts occurring during PCR amplification, incorrect local alignment or problems due to tumor heterogeneity [16]. According to the data presented here, concordance of sequencing results with different variant calling pipelines was generally low. A pipeline based on Unified Genotyper by GATK and SnpEff was used and compared with the output of SAMtools/BCFtools and the Illumina Somatic Variant Caller regarding both FFPE and frozen samples in direct comparison. The former appeard to be less sensitive than Unified Genotyper pipeline, however, it also identified variants not found by the other pipelines. The latter method showed more variant calls than the other pipelines. However, since many variant calls were present either only in the FFPE or the frozen sample of the same patient, and since several in CRC unexpected or unusual mutations appeared, especially in poor quality samples, it is very likely that many of the additional variants identified by this pipeline are false positive. Compared with Sanger sequencing of KRAS exon 2, a high concordance of Unified Genotyper pipeline results was shown. For one patient, we observed a discordant mutation status by Sanger and Illumina sequencing. However, Sanger sequencing was performed with material from the primary tumor and the sample from the liver metastsis had an estimated tumor content of only 10%. Notably, no mutant reads were observed in at the KRAS exon 2 locus in the deep sequencing data. Hence, tumor heterogeneity or low tumor content of the sequenced material can have led to the false-negative result. Other groups have analyzed concordance of different variant calling pipelines in whole-exome and whole-genome data [7,16–18]. O’Rave et al. [7] sequenced 15 exomes and found a low concordance of only 57% for SNPs running five different analysis pipelines and 27% for Indels running three different analysis pipelines with near default parameters. Pabinger et al. [17] provided a broad overview on software for NGS data analysis and tested 32 different programs for variant identification, annotation and visualization on four data sets including two cancer datasets. They grouped tools into such categories as “germline callers” and “somatic callers”. The concordance of five tested “germline callers” was low for SNPs and zero for Indels, while they also found no common variant with three “somatic callers”, analyzing whole-exome datasets. These studies highlight the problems of accurate variant identification in large-scale whole-exome data. Our study, however, is to our knowledge the first to raise this issue in rather small-scale, targeted amplicon sequencing data from clinical, formalin-fixed samples. Some authors suggested to analyze datasets with different variant callers and to combine their results. However, comparing the results of different tools that apply different quality metrics, can be difficult and time consuming. Statistical methods, such as applying false-discovery-rate confidence values, have been developed to rank mutation calls from different tools [19]. More elaborate experimental procedures like sequencing replicates of normal tissue are necessary for such methods, which reduce feasibility of such methods for clinical amplicon sequencing, for which in many retrospective settings not even normal tissue is available as a reference. The same holds true for extensive validation of data by applying different sequencing technologies, e.g. Sanger sequencing, to rule out false-positive calls. This may in our view be a feasible approach to confirm novel SNPs correlated to inherited diseases in whole-exome studies of individual patients, but not for the study of cancer genomes in retrospective studies on limited archival tumor material. Moreover, the problem of false negatives cannot be overcome with this approach.

The study of FFPE material makes analysis even more difficult due to highly fragmented DNA and SNP artifacts, for instance related to cytosine deamination to uracil [20]. Our data indicate general feasibility of amplicon sequencing using FFPE tissue, demonstrating a high concordance of variant calls in matched frozen and FFPE samples using GATK Unified Genotyper pipeline with adapted filter criteria. Notably, the two non-matching mutations (BRAF V600E and ATM E1971G) were both identified in the FFPE sample but not in the frozen sample of the same patient, suggesting false positive calls due to low DNA quality (FFPE sample of patient 09 had comparably high **Δ**Cq value, comp. S2 Table). Sanger sequencing of the BRAF mutational hotspot in exon 15, however, revealed V600E mutation. Hence, variant calling of amplicon sequencing led to a false negative result in frozen tissue, most likely due to low amounts of tumor cells or tumor heterogeneity. Interestingly, six per cent mutated reads of >10,000 reads at the BRAF V600E locus in the frozen sample were not sufficient for a mutation call by Unified Genotyper pipeline.

Substantiating our results, a few authors have reported NGS studies using FFPE material, also demonstrating feasibility of this approach [6,21–28]. For instance, Wagle et al. [26] successfully applied exome capture sequencing to target 137 “druggable” mutations in 10 FFPE samples form colon and breast cancer patients. Spencer et al. [6] provided the yet only study directly comparing paired frozen and FFPE samples from 16 patients with lung cancer, using hybridization capture enrichment for sequencing 27 cancer related genes. They also used Unified Genotyper from GATK for variant calling. They also found greater coverage variability and increased C to T transitions in FFPE samples while base calls between paired frozen and FFPE samples had concordances as high as 99%.

An important issue for sequencing of FFPE samples is DNA preparation, qualification and library preparation [29]. It is recommended to measure DNA with Qubit (and NanoDrop) assays to assess purity and quantity of DNA [29]. As in previous reports, quantity of DNA isolated from our samples measured by NanoDrop differed from Qubit results, with NanoDrop generally overestimating DNA amounts [29]. Moreover, it has been recommended to quantify the “functional”, amplifiable DNA content of DNA isolated from FFPE tissue before applying to NGS techniques, especially to PCR based amplicon sequencing. Sah et al. [30] reported that a qPCR based assay (QFI-PCR), similar to the FFPE quality assay (Illumina) that we performed on our samples, could identify poorest quality samples. Moreover, similar to our approach, samples with low amounts of amplifiable DNA could be “rescued” by increasing input amounts. According to our data, the amount of amplifiable DNA (represented by a low **Δ**Cq-Value) in FFPE samples correlates with the amount of properly amplified library DNA. We also could rescue some of the samples that had libraries with low amounts of properly amplified DNA by increasing DNA input. Since we used maximum DNA input, the ideal increase in DNA amount for poor samples and also the cut-off to exclude poorest samples remains to be defined. This is especially valid as a minimum input of precious DNA as possible is desired. Interestingly, the samples with lowest amounts of amplifiable DNA also had a higher number of variant calls and a markedly increased number of false positive calls according to data from Sah et al. [30], indicating that the amount of amplifiable DNA is also a surrogate for general DNA quality. In our dataset, similar effects could be observed. The depth of sequencing was lower for samples with high **Δ**Cq-values. Moreover, samples with low amounts of properly amplified library DNA, as measured by Bioanalyzer, tended to have many (most likely false-positive) variant calls, when analyzed with Somatic Variant Caller pipeline (compare S2 Table, Fig 4, S5 Table). However, with the Unified Genotyper variant calling pipeline and strict variant filtering, this effect could be diminished. Thus, poor sample quality can in part be compensated with advanced bioinformatics methods. Nevertheless, this pipeline appears to have the problem of enhanced false negatives in samples with poor quality compared to the Somatic Variant Caller pipeline (compare Fig 4), indicating that variant calling remains problematic especially in samples with poor DNA quality. In any case, best possible sample preparation is crucial to allow optimal results in variant calling. Methods for biochemical modification of DNA from FFPE tissues have been proposed [20], however, more data is needed to verify these interesting results before implementing into current practice. Criteria for excluding poor quality samples from sequencing also have to be refined. A recommended cut-off of **Δ**Cq >2 would have excluded almost half of our samples, which is not satisfactory for clinical studies with rare patient material. Further, larger studies would need to be employed to identify potentially suitable cut-off values. Remarkably, large differences exist between the recommended amounts of input DNA between Illumina TruSeq amplicon panel (>250ng) and IonTorrent AmpliSeq panel, for which libraries can be prepared with as little as 10ng DNA.

Our study has several limitations. First, only a small set of patients has been analyzed. Larger series of FFPE samples have to be sequenced to show feasibility for routine practice and clinical studies. Moreover, many other variant calling algorithms and pipelines are available, that are steadily improving. Nevertheless, we believe that the problems of reproducibility of variant calling can be well demonstrated with this small dataset of matched frozen and FFPE samples, exemplified by the tested variant calling pipelines. Almost all of our samples were from 2012. A larger amount of older FFPE samples would have to be analyzed to define their usability for clinical sequencing. Data from previous studies, however, suggests that age does not generally have a big impact on DNA quality, but rather fixation time in formalin seems to be of major importance [6].

## Conclusions

In conclusion, our data shows that amplicon sequencing of clinical CRC samples is a viable approach to characterize druggable, predictive or prognostic mutations in the cancer genome. A high concordance between mutations identified in frozen tissue and paired FFPE samples does furthermore suggest that also archived tissues from pathology departments can be used for genomic profiling with this method. However, bioinformatic pipelines for data analysis yet show marked differences in results. Moreover, dedicated sample and library preparation and qualification, including exclusion of poorest quality samples, have to be done. For the use of amplicon sequencing in routine diagnostics or in clinical studies, gold standard methods have to be defined, which should lead to higher reproducibility.

## Supporting Information

S1 Fig Sequencing libraries produced with low-quality DNA. Bioanalyzer was used to measure amounts of DNA by fragment length of DNA from FFPE samples during the library preparation workflow. Three representative samples are shown: Patient 04 had high levels of DNA with the aspired DNA fragment size of ~310bp and low amounts of short length DNA fragments <100bp after initial library preparation with standard input amounts. Patients 13 and 15 are examples for low quality DNA with low amounts of DNA around 310bp and high amounts of highly fragmented DNA. Library preparation was repeated for those samples using maximum DNA input (compare S2 Table), which led to significantly higher concentrations of correct sized DNA for sample 13, but not for sample 15. Highly increased background, short-fragment DNA was shown to be reduced after the PCR clean-up step. Patient 13 was then sequenced, while patient 15 was excluded.
(TIF)

S2 Fig Functional DNA amount correlates with GC-content. (A) ΔCq-values of quality control PCR of FFPE samples are plotted against GC content of sample DNA. (B) GC content of sample DNA and mean depth of sequencing of FFPE samples analyzed.
(TIF)

S3 Fig No KRAS mutation identified by amplicon sequencing in patient 02. Representative image of reads mapped to the site of KRAS exon 2 with no mutated reads detected at the mutational hot-spot at codon 38 in FFPE tissue from the liver metastasis, displayed with the Integrative Genomics Viewer. KRAS mutation had been detected in the primary tumor by Sanger sequencing. The expected mutational locus is indicated by black lines.
(TIF)

S4 Fig Filtering removes false positive variant calls in cell lines analyzed by deep amplicon sequencing. (A,B) Variant calling of deep amplicon sequencing data from cell lines HCT116, DLD-1, SW480, HUH7, HT55, HEK293T and HS68 was performed with GATK Unified Genotyper (UG), SamTools/BcfTools (SAM) or Illumina Somatic Variant Caller (SVC) without any filtering of variants (A) or with exclusion of variants below defined quality thresholds, synonymous and non-coding variants, as well as variants present in the 1000G data (B). Concordance of genomic variant loci identified with the tree pipelines was analyzed with jvenn. (C,D) Overlap of variant loci identified in HCT116, HT55, HUH7 and SW480 with the GATK Unified Genotyper pipeline with variant loci detected by the Cell Line Encyclopedia Project [13] is shown without (C) or with (D) filtering out variants below quality thresholds, synonymous and non-coding variants, as well as variants present in the 1000G data. (E,F) Overlap of variant loci identified in HCT116, HT55 and HUH7 with the GATK Unified Genotyper pipeline with variant loci detected by the COSMIC cell line project [14] is shown without (E) or with (F) filtering out low quality variants, synonymous and non-coding variants, as well as non-harmful variants present in the 1000G data.
(TIF)

S5 Fig Concordance of variant loci in frozen and FFPE samples analyzed with three different variant calling pipelines with and without filtering. (A) Variant calling of sequencing data from matched frozen and FFPE samples were performed with GATK Unified Genotyper (UG), SamTools/BcfTools (SAM) or Illumina Somatic Variant Caller (SVC) without any filtering of variants. Overlap of genomic variant loci identified in each group are shown. Below, the number of variant loci identified in each group are outlined. (B) Variants from (A) were annotated and variants with low quality metrics, synonymous and non-coding variants, as well as variants present in the 1000G data were filtered out. Again, overlap of genomic variant loci identified in each group are shown. Below, the number of variant loci identified in each group are outlined. Fields with “0” overlap are left empty.
(TIF)

S6 Fig Variant calling is more sensitive when samples are processed together compared with analyzing each sample individually. (A, B) GATK Unified Genotyper pipeline with variant calling in all analyzed samples together or (C, D) separate. Green color represents FFPE samples, red represents frozen, color intensities represent number of non-synonymous coding mutations per gene.
(TIF)

S1 Table Patients. (PDF)

S2 Table Sample and library preparation. (PDF)

S3 Table List of amplicons and targeted regions. (XLSX)

S4 Table Patient sequencing statistics. (XLSX)

S5 Table Mutations identified in primary tumors with different variant calling pipelines. (PDF)

S6 Table Mutations identified in cell lines with different variant calling pipelines. (PDF)

S7 Table All variants identified in analyzed samples (XLSX)

S8 Table All unfiltered variants identified in analyzed samples (XLSX)
