# Supplementary figures and images for: Amplicon Sequencing of Colorectal Cancer: Variant Calling in Frozen and Formalin-Fixed Samples
Source: PLoS One. 2015 May 26;10(5):e0127146. doi: 10.1371/journal.pone.0127146 (PMC4444292; doi:10.1371/journal.pone.0127146)

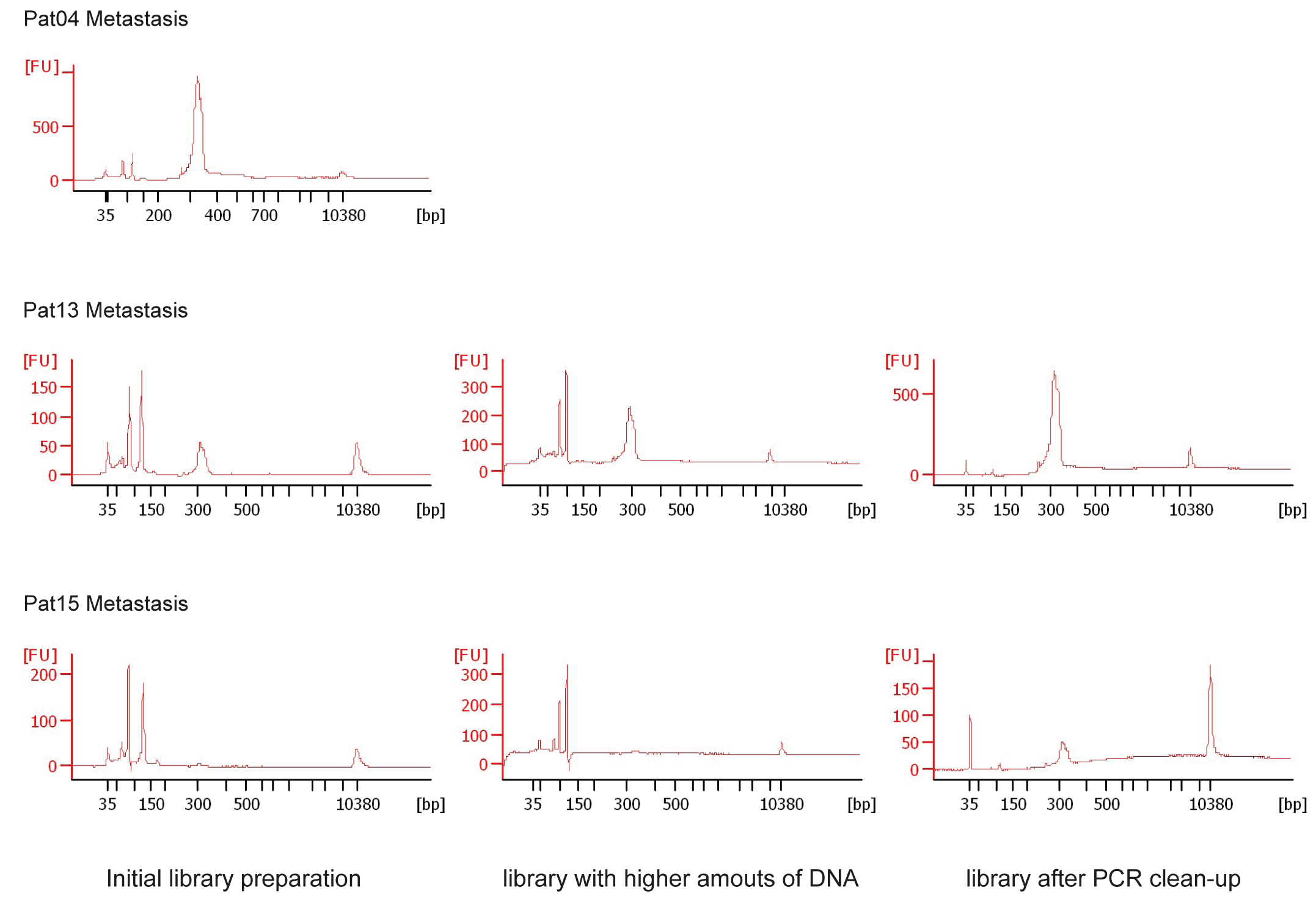

Supplement: S1 Fig — Bioanalyzer was used to measure amounts of DNA by fragment length of DNA from FFPE samples during the library preparation workflow. Three representative samples are shown: Patient 04 had high levels of DNA with the aspired DNA fragment size of ~310bp and low amounts of short length DNA fragments <100bp after initial library preparation with standard input amounts. Patients 13 and 15 are examples for low quality DNA with low amounts of DNA around 310bp and high amounts of highly fragmented DNA. Library preparation was repeated for those samples using maximum DNA input (compare S2 Table), which led to significantly higher concentrations of correct sized DNA for sample 13, but not for sample 15. Highly increased background, short-fragment DNA was shown to be reduced after the PCR clean-up step. Patient 13 was then sequenced, while patient 15 was excluded. (TIF) [file pone.0127146.s001.tif]

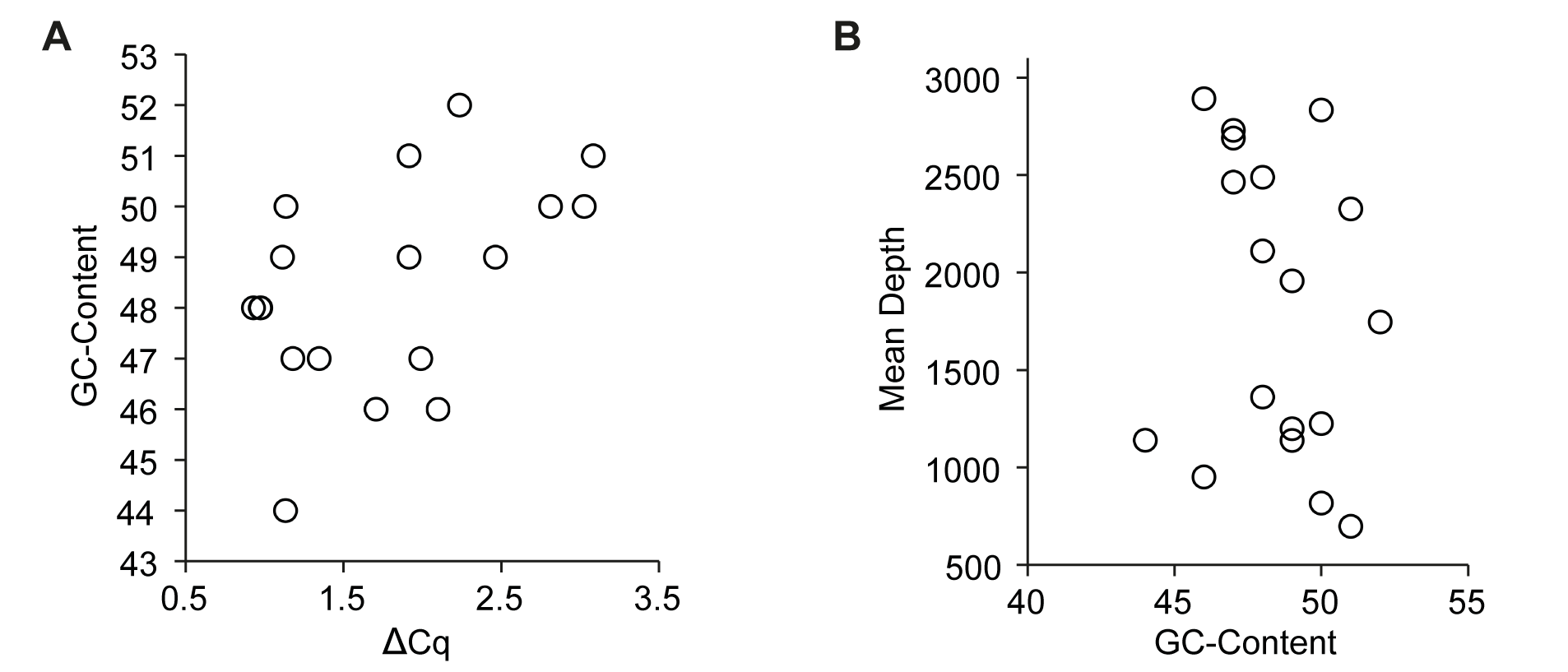

Supplement: S2 Fig — (A) ΔCq-values of quality control PCR of FFPE samples are plotted against GC content of sample DNA. (B) GC content of sample DNA and mean depth of sequencing of FFPE samples analyzed. (TIF) [file pone.0127146.s002.tif]

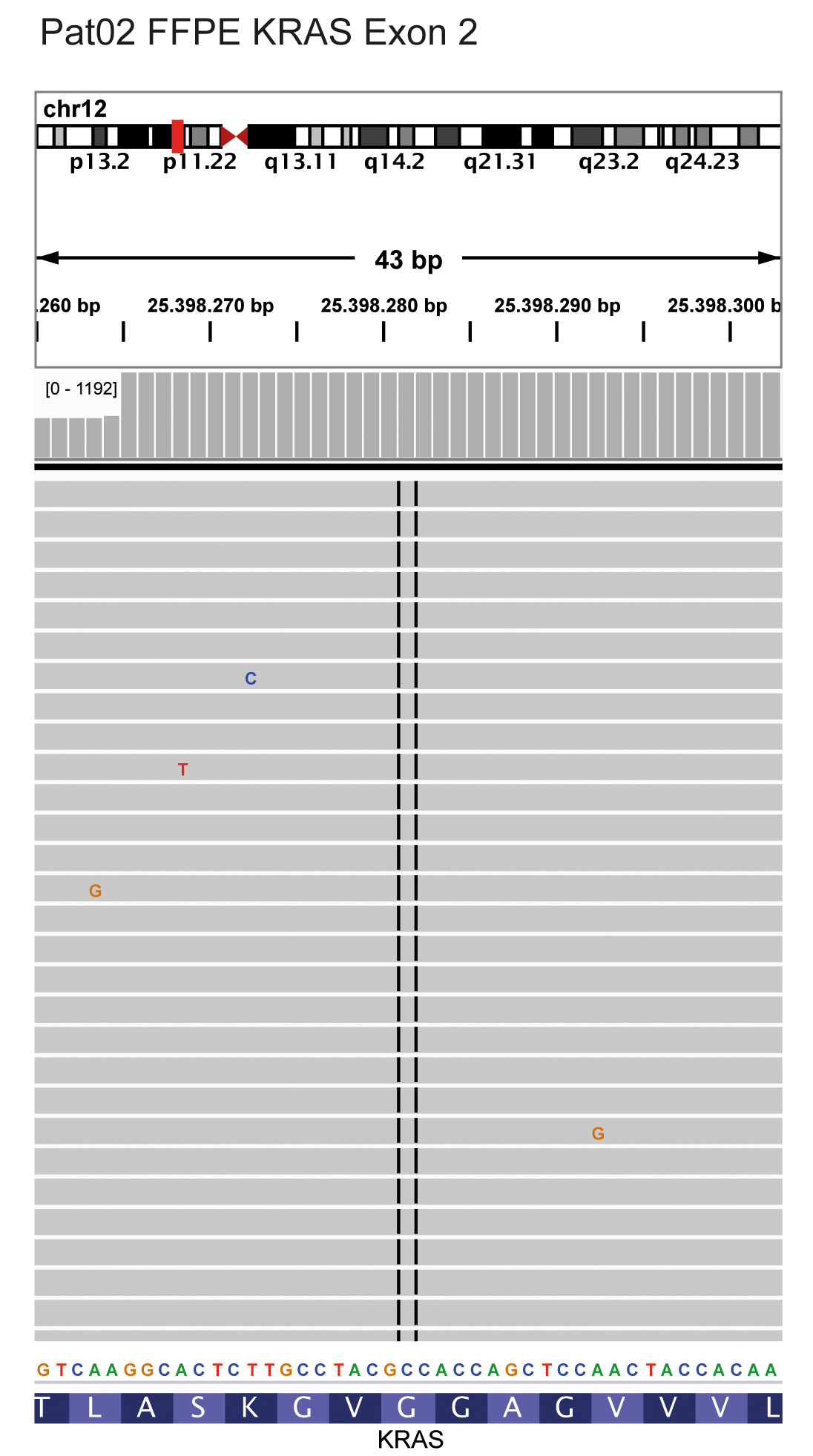

Supplement: S3 Fig — Representative image of reads mapped to the site of KRAS exon 2 with no mutated reads detected at the mutational hot-spot at codon 38 in FFPE tissue from the liver metastasis, displayed with the Integrative Genomics Viewer. KRAS mutation had been detected in the primary tumor by Sanger sequencing. The expected mutational locus is indicated by black lines. (TIF) [file pone.0127146.s003.tif]

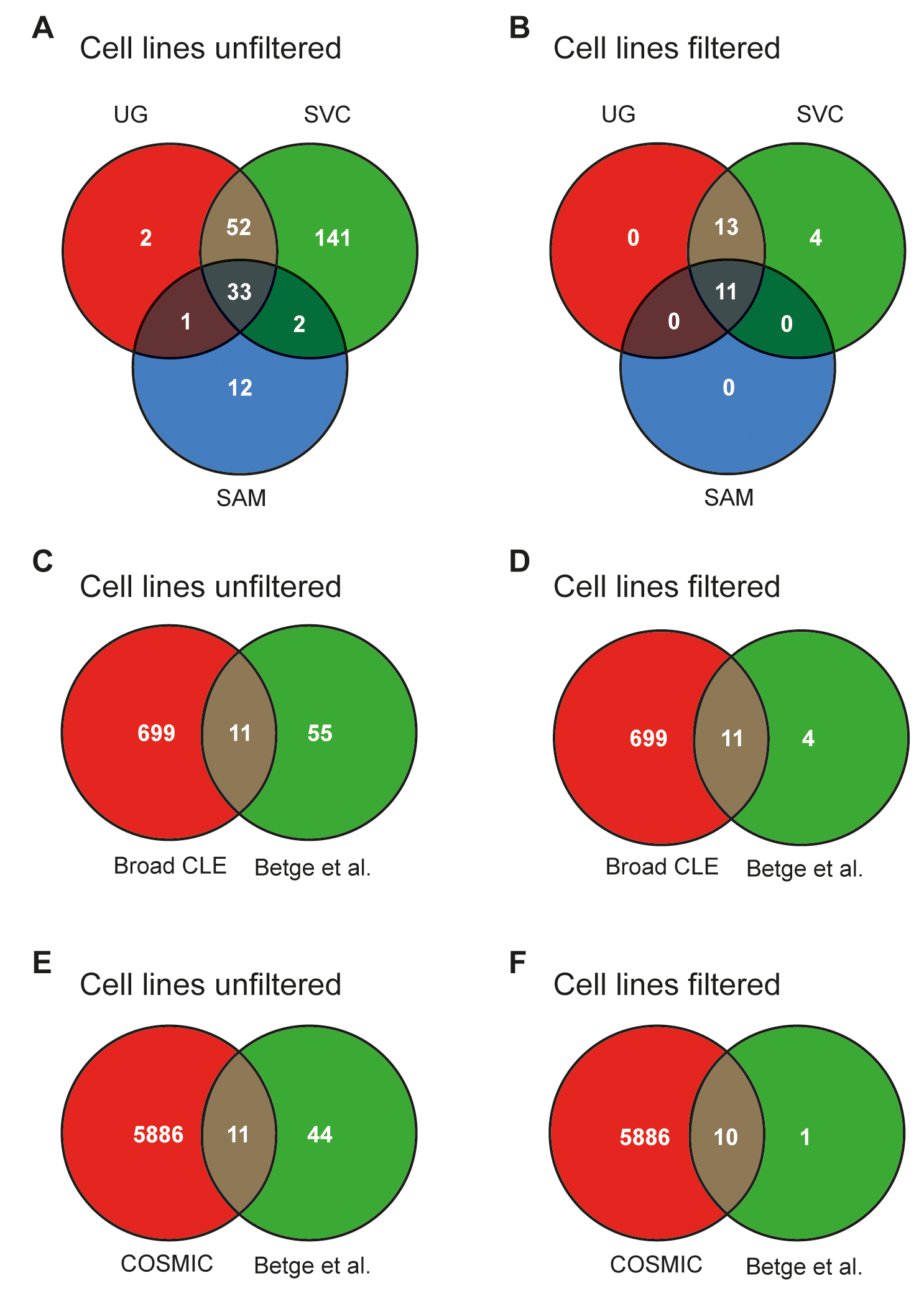

Supplement: S4 Fig — (A,B) Variant calling of deep amplicon sequencing data from cell lines HCT116, DLD-1, SW480, HUH7, HT55, HEK293T and HS68 was performed with GATK Unified Genotyper (UG), SamTools/BcfTools (SAM) or Illumina Somatic Variant Caller (SVC) without any filtering of variants (A) or with exclusion of variants below defined quality thresholds, synonymous and non-coding variants, as well as variants present in the 1000G data (B). Concordance of genomic variant loci identified with the tree pipelines was analyzed with jvenn. (C,D) Overlap of variant loci identified in HCT116, HT55, HUH7 and SW480 with the GATK Unified Genotyper pipeline with variant loci detected by the Cell Line Encyclopedia Project [13] is shown without (C) or with (D) filtering out variants below quality thresholds, synonymous and non-coding variants, as well as variants present in the 1000G data. (E,F) Overlap of variant loci identified in HCT116, HT55 and HUH7 with the GATK Unified Genotyper pipeline with variant loci detected by the COSMIC cell line project [14] is shown without (E) or with (F) filtering out low quality variants, synonymous and non-coding variants, as well as non-harmful variants present in the 1000G data. (TIF) [file pone.0127146.s004.tif]

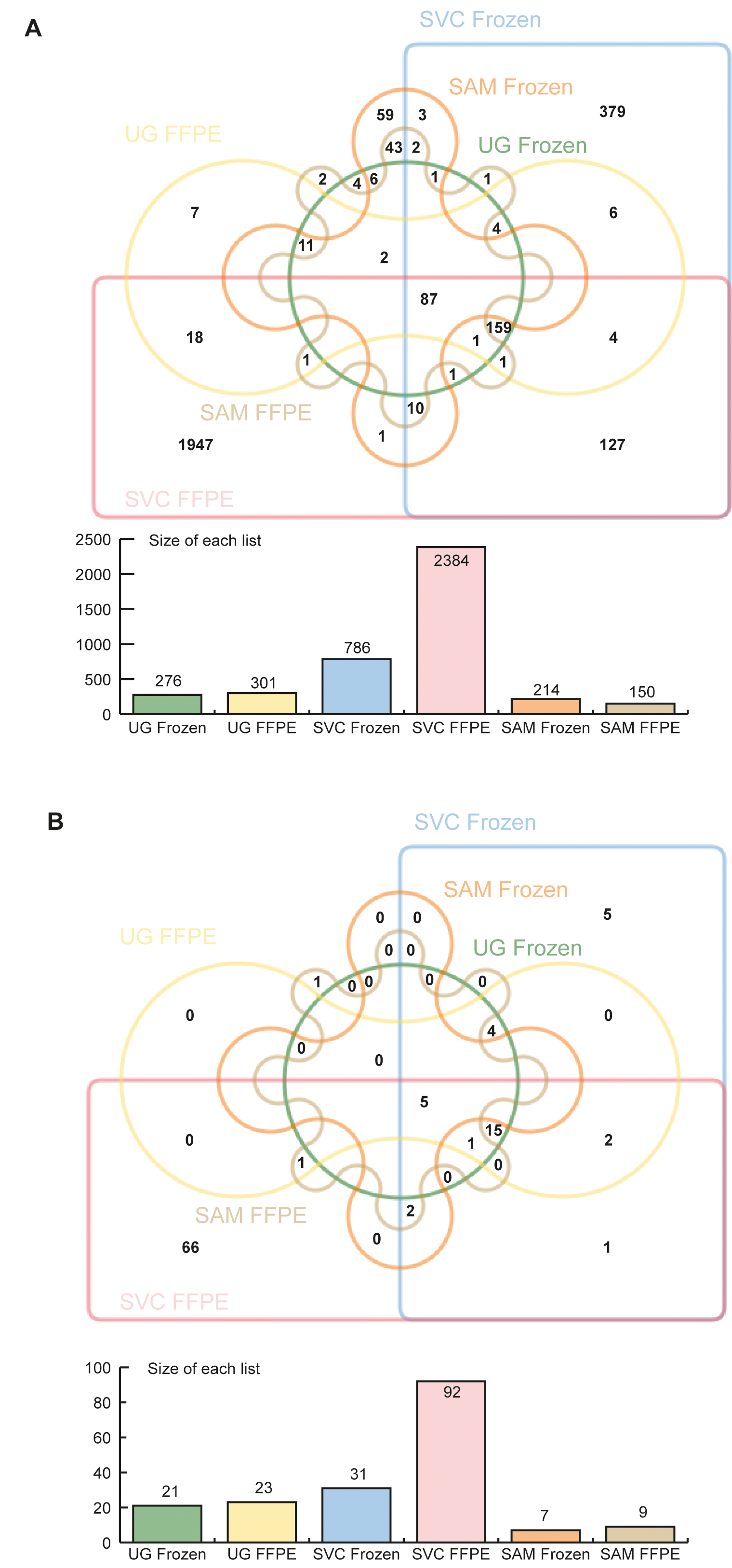

Supplement: S5 Fig — (A) Variant calling of sequencing data from matched frozen and FFPE samples were performed with GATK Unified Genotyper (UG), SamTools/BcfTools (SAM) or Illumina Somatic Variant Caller (SVC) without any filtering of variants. Overlap of genomic variant loci identified in each group are shown. Below, the number of variant loci identified in each group are outlined. (B) Variants from (A) were annotated and variants with low quality metrics, synonymous and non-coding variants, as well as variants present in the 1000G data were filtered out. Again, overlap of genomic variant loci identified in each group are shown. Below, the number of variant loci identified in each group are outlined. Fields with “0” overlap are left empty. (TIF) [file pone.0127146.s005.tif]

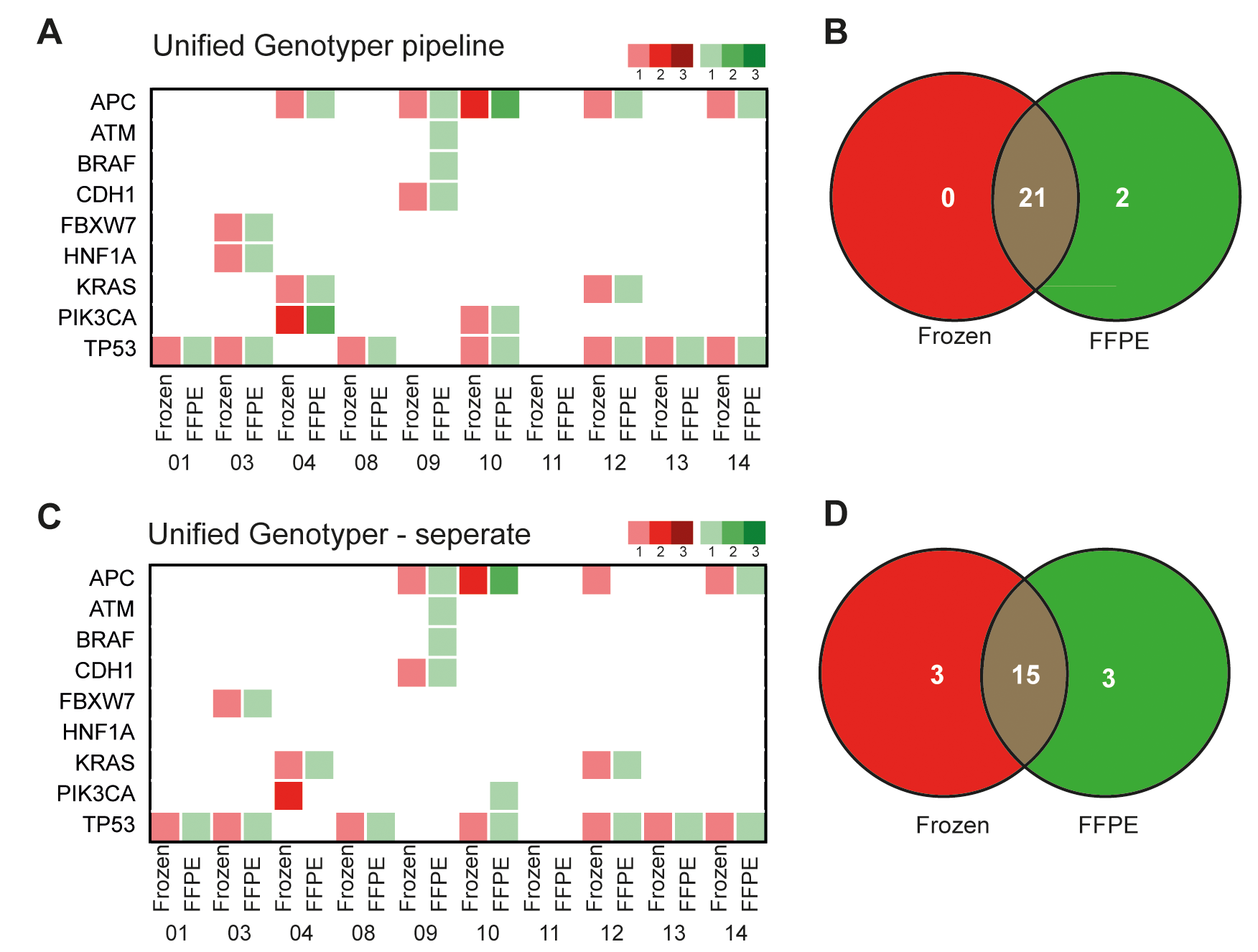

Supplement: S6 Fig — (A, B) GATK Unified Genotyper pipeline with variant calling in all analyzed samples together or (C, D) separate. Green color represents FFPE samples, red represents frozen, color intensities represent number of non-synonymous coding mutations per gene. (TIF) [file pone.0127146.s006.tif]
