# Supplementary material for: Amplicon Sequencing of Colorectal Cancer: Variant Calling in Frozen and Formalin-Fixed Samples
Source: PLoS One. 2015 May 26;10(5):e0127146. doi: 10.1371/journal.pone.0127146 (PMC4444292; doi:10.1371/journal.pone.0127146)
Supplement: S1 Table — (PDF) [file pone.0127146.s007.pdf]

S1 Table. Patients

Patient No.	Sex	Age	Histo	FFPE analyzed	Frozen analyzed	Primary analyzed
Pat01	M	59	CRC	yes	yes	no
Pat02	F	58	CRC	yes	no	yes
Pat03	F	59	CRC	yes	yes	no
Pat04	M	63	CRC	yes	yes	yes
Pat05	M	65	NET	yes	no	yes
Pat06	M	47	CRC	yes	no	no
Pat07	F	53	CRC	yes	no	no
Pat08	M	74	CRC	yes	yes	no
Pat09	M	66	CRC	yes	yes	no
Pat10	M	50	CRC	yes	yes	yes
Pat11	M	69	CRC	yes	yes	yes
Pat12	M	47	CRC	yes	yes	no
Pat13	M	56	CRC	yes	yes	no
Pat14	M	70	CRC	yes	yes	yes
Pat15	M	52	CRC	yes	yes	no
Pat16	M	63	CRC	no	yes	no
Pat17	F	49	CRC	no	yes	no
Pat18	M	70	CRC	no	yes	no
