# Supplementary material for: Amplicon Sequencing of Colorectal Cancer: Variant Calling in Frozen and Formalin-Fixed Samples
Source: PLoS One. 2015 May 26;10(5):e0127146. doi: 10.1371/journal.pone.0127146 (PMC4444292; doi:10.1371/journal.pone.0127146)
Supplement: S2 Table — (PDF) [file pone.0127146.s008.pdf]

S2 Table. Sample and library preparation

A Sample and library preparation

Patient	Tumor	Site	Elution volume (µl)	Sample Concentration Nanodrop (ng/µl)	OD 260/280	Sample Concentration Qubit (ng/µl)	ΔCq	Bioanalyzer concentration of 250-460bp Fragments (pg/µl)
Pat01	CRC	Met	40	1023	1.78	385	1.1	2552.2
Pat02	CRC	Met	40	296	1.79	120	1.9	519.0
Pat02	CRC	Prim	40	178	1.81	87	3.5	343.7
Pat03	CRC	Met	40	1144	1.83	570	1.2	1727.9
Pat04	CRC	Met	40	612	1.86	263	0.9	6272.3
Pat04	CRC	Prim	40	572	1.82	250	2.8	211.3
Pat05	NET	Met	40	775	1.79	371	1.7	93831.9
Pat05	NET	Prim	40	702	1.79	331	1.3	1270.0
Pat06	CRC	Met	40	130	1.78	43	1.1	2944.4
Pat07	CRC	Met	40	385	1.78	200	3.4	137.7
Pat08	CRC	Met	40	1008	1.81	494	1.1	1445.2
Pat09	CRC	Met	40	300	1.81	155	3.1	560.5
Pat10	CRC	Met	40	411	1.86	194	1.0	2385.8
Pat10	CRC	Prim	40	355	1.85	146	2.1	2125.6
Pat11	CRC	Met	40	460	1.84	185	1.0	1186.6
Pat11	CRC	Prim	40	400	1.85	181	2.0	334.3
Pat12	CRC	Met	40	493	1.8	271	3.0	93.3
Pat13	CRC	Met	40	823	1.79	400	2.2	289.6
Pat14	CRC	Met	40	437	1.84	196	1.9	672.2
Pat14	CRC	Prim	40	690	1.78	285	2.5	222.1
Pat15	CRC	Met	40	480	1.77	247	4.1	51.7

B Repeated library preparation of poor quality samples

Patient	Tumor	Site	DNA used for Library preparation (ng)	Bioanalyzer DNA concentration 250-450bp	Bioanalyzer DNA concentration 250-450bp after PCR clean-up	Sequenced
Pat01	CRC	Met	1540	5713.62	5118.85	yes
Pat02	CRC	Met	1080	2899.94	1265.03	yes
Pat02	CRC	Prim	2175	1902.77	451.17	no
Pat04	CRC	Prim	6250	1501.10	891.13	yes
Pat07	CRC	Met	5000	365.34	160.25	no
Pat09	CRC	Met	3875	1829.95	1148.27	yes
Pat11	CRC	Prim	1629	5669.87	3937.40	yes
Pat12	CRC	Met	6775	1979.41	1233.89	yes
Pat13	CRC	Met	10000	41821.50	2222.69	yes
Pat14	CRC	Met	1764	7244.96	3322.90	yes
Pat14	CRC	Prim	7125	2013.29	2423.12	yes
Pat15	CRC	Met	6175	1348.61	130.63	no
