# Supplementary material for: Amplicon Sequencing of Colorectal Cancer: Variant Calling in Frozen and Formalin-Fixed Samples
Source: PLoS One. 2015 May 26;10(5):e0127146. doi: 10.1371/journal.pone.0127146 (PMC4444292; doi:10.1371/journal.pone.0127146)
Supplement: S5 Table — (PDF) [file pone.0127146.s011.pdf]

S5 Table. Mutations in primary tumors

	Unified Genotyper, SnpEff						Samtools mpileup/Bcftools, SnpEff						Somatic Variant Caller					
	Chr	Pos	Ref	Alt	Gene	AA-Change	Chr	Pos	Ref	Alt	Gene	AA-Change	Chr	Pos	Ref	Alt	Gene	AA-Change
Pat04													1	115258757	C	T	NRAS	V9I
													2	29432734	C	T	ALK	A1252T
													2	212288982	C	T	ERBB4	D922N
													2	212589856	C	T	ERBB4	C229Y
													2	212589892	C	T	ERBB4	C217Y
													3	178916893	C	T	PIK3CA	L94F
													3	178936082	G	A	PIK3CA	E542K
													3	178936094	C	T	PIK3CA	Q546X
	3	178938934	G	A	PIK3CA	E726K							3	178938934	G	A	PIK3CA	E726K
													3	178951946	C	T	PIK3CA	L1001F
													4	1803571	C	T	FGFR3	P250L
													4	1806186	C	T	FGFR3	P402L
													4	1806233	C	T	FGFR3	P418S
													4	1808320	C	T	FGFR3	S693F
													4	55144167	A	C	PDGFRA	K666Q
													4	55144650	TG	T	PDGFRA	Frameshift
													4	55561861	C	T	KIT	T84M
													4	55593414	C	T	KIT	P520L
													4	55593600	C	T	KIT	Q552X
													4	55597529	C	T	KIT	P722L
													4	55597550	CA	C	KIT	Frameshift
													4	55955117	C	T	KDR	W1143X
													2	212288940	C	T	ERBB4	G936R
													4	153245528	C	T	FBXW7	V475M
													2	212578365	C	T	ERBB4	V298M
													4	153249421	A	T	FBXW7	C373S
													4	153259081	C	T	FBXW7	S165N
													5	112175112	G	A	APC	C1256Y
													5	112175151	T	A	APC	I1269K
													5	112175154	G	A	APC	G1270E
	5	112175211	TAAAAG	T	APC	Frameshift	5	112175211	TAAAAGAAAAGA	TAAAAGA	APC	Frameshift	5	112175211	TAAAAG	T	APC	Frameshift
													5	112175558	C	T	APC	L1405F
													5	112175589	C	T	APC	P1415L
													5	112175612	C	T	APC	P1423S

			5	112175616	C	T	APC	P1424L
			5	112175681	G	A	APC	E1446K
			5	170837543	C	T	NPM1	L258F
			7	55259533	C	T	EGFR	A864V
			7	116411990	C	T	MET	T1010I
			7	128846398	C	T	SMO	L412F
			4	55593584	A	T	KIT	K546N
			7	128851594	C	T	SMO	T640I
			7	140453137	C	T	BRAF	V600M
			7	140453167	C	T	BRAF	V590I
			9	80412476	TC	T	GNAQ	Frameshift
			4	153245443	A	T	FBXW7	L503X
			9	133748382	C	T	ABL1	S367L
			4	153247304	G	T	FBXW7	H420N
			10	89624246	A	T	PTEN	E7V
			10	89720851	C	A	PTEN	N334K
			5	112174747	G	T	APC	Q1134H
			10	123274807	C	T	FGFR2	A259T
			10	123279627	C	T	FGFR2	V154M
			10	123279654	C	T	FGFR2	A145T
			11	108117852	C	T	ATM	Q355X
			11	108123557	C	T	ATM	L606F
			11	108172482	C	T	ATM	A1762V
			11	108172490	C	T	ATM	Q1765X
			11	108180964	C	T	ATM	S1947F
			11	108180985	C	T	ATM	A1954V
			11	108236212	C	T	ATM	P3050S
			12	25378673	C	T	KRAS	V109I
			12	25380325	C	T	KRAS	V45I
			12	25398284	C	T	KRAS	G12D
			12	25398300	C	T	KRAS	V7M
			13	28592644	C	T	FLT3	R834Q
			13	28602326	C	T	FLT3	C681Y
			13	28608247	C	T	FLT3	W603X
			13	28608264	C	T	FLT3	E598K
			9	139397766	C	T	NOTCH1	E1679K
			13	49039230	C	T	RB1	Q770X
			16	68846159	C	T	CDH1	P377L
			17	7578179	C	T	TP53	E92K
			17	7578263	G	A	TP53	R64X
			17	7578272	G	A	TP53	H61Y

													17	7579364	C	T	TP53	G108D
													18	48581246	C	T	SMAD4	H184Y
													18	48584572	C	T	SMAD4	Q249X
													18	48591904	C	T	SMAD4	P356L
													18	48603113	C	T	SMAD4	P472S
													18	48604760	C	T	SMAD4	H528Y
													19	1207030	C	T	STK11	R40C
													19	1207039	G	T	STK11	A43S
													19	3114961	C	A	GNA11	R166S
													19	3118924	T	A	GNA11	M203K
													19	3118929	G	A	GNA11	D205N
													12	121432118	C	T	HNF1A	P289S
													12	121432187	C	T	HNF1A	L312F
													19	3118935	G	A	GNA11	G207R
													22	24133995	C	T	SMARCB1	S49L
													22	24134049	C	T	SMARCB1	S67L
													22	24134051	C	T	SMARCB1	H68Y
Pat10													3	178952085	A	G	PIK3CA	H1047R
													5	112175171	C	T	APC	Q1276X
													5	112175639	C	T	APC	R1432X
													17	7578406	C	T	TP53	R43H
Pat11													4	153247285	C	T	FBXW7	C426Y
													11	108201014	C	T	ATM	R2461C
													12	121432119	C	T	HNF1A	P289L
													12	121432175	C	T	HNF1A	P308S
Pat14	5	112175245	TC	T	APC	Frameshift							5	112175245	TC	T	APC	Frameshift
	17	7578263	G	A	TP53	R196*	17	7578263	G	A	TP53	R196*	17	7578263	G	A	TP53	R64X
