# Supplementary material for: Amplicon Sequencing of Colorectal Cancer: Variant Calling in Frozen and Formalin-Fixed Samples
Source: PLoS One. 2015 May 26;10(5):e0127146. doi: 10.1371/journal.pone.0127146 (PMC4444292; doi:10.1371/journal.pone.0127146)
Supplement: S6 Table — (PDF) [file pone.0127146.s012.pdf]

S6 Table. Mutations in celllines

	Unified Genotyper, SnpEff						Samtools mpileup/Bcftools, SnpEff						Somatic Variant Caller					
	Chr	Pos	Ref	Alt	Gene	AA-Change	Chr	Pos	Ref	Alt	Gene	AA-Change	Chr	Pos	Ref	Alt	Gene	AA-Change
DLD1	2	209113217	C	T	IDH1	G97D							2	209113217	C	T	IDH1	G97D
DLD1	3	178936091	G	A	PIK3CA	E545K							3	178936091	G	A	PIK3CA	E545K
DLD1	3	178936103	G	A	PIK3CA	D549N	3	178936103	G	A	PIK3CA	D549N	3	178936103	G	A	PIK3CA	D549N
DLD1	4	55593437	G	A	KIT	V528I	4	55593437	G	A	KIT	V528I	4	55593437	G	A	KIT	V528I
DLD1	5	112175538	GC	G	APC	FRAMESHIFT							5	112175538	GC	G	APC	FRAMESHIFT
DLD1													5	112175907	C	T	APC	S1521L
DLD1	7	116339688	G	T	MET	V184F							7	116339688	G	T	MET	V184F
DLD1													7	128851593	A	G	SMO	T640A
DLD1	9	139399401	G	A	NOTCH1	P1581L	9	139399401	G	A	NOTCH1	P1581L	9	139399401	G	A	NOTCH1	P1581L
DLD1	12	25398281	C	T	KRAS	G13D							12	25398281	C	T	KRAS	G13D
DLD1	17	7577559	G	A	TP53	S109F	17	7577559	G	A	TP53	S109F	17	7577559	G	A	TP53	S109F
DLD1	19	1223061	G	A	STK11	R333H							19	1223061	G	A	STK11	R333H
HCT116	3	41266133	CCTT	C	CTNNB1	PS44P	3	41266133	CCTTCT	CCT	CTNNB1	S45-	3	41266133	CCTT	C	CTNNB1	Deletion
HCT116	3	178952085	A	G	PIK3CA	H1047R							3	178952085	A	G	PIK3CA	H1047R
HCT116	7	128846374	G	A	SMO	V404M	7	128846374	G	A	SMO	V404M	7	128846374	G	A	SMO	V404M
HCT116	9	133738370	A	G	ABL1	Y257C	9	133738370	A	G	ABL1	Y257C	9	133738370	A	G	ABL1	Y276C
HCT116	12	25398281	C	T	KRAS	G13D							12	25398281	C	T	KRAS	G13D
HCT116	12	121432114	CG	C	HNF1A	FRAMESHIFT							12	121432114	CG	C	HNF1A	FRAMESHIFT
HT55	5	112174682	C	T	APC	Q1113*							5	112174682	C	T	APC	Q1113X
HT55	5	112175198	C	T	APC	Q1285*							5	112175198	C	T	APC	Q1285X
HT55							5	112175680	A	T	APC	R1445S	5	112175680	A	T	APC	R1445S
HT55	17	7578211	C	A	TP53	R213L							17	7578211	C	A	TP53	R81L
HUH7	17	7578190	T	C	TP53	Y220C	17	7578190	T	C	TP53	Y220C	17	7578190	T	C	TP53	Y88C
HEK293T	12	121432114	CG	C	HNF1A	FRAMESHIFT							12	121432114	CG	C	HNF1A	FRAMESHIFT
HEK293T	17	7577098	T	A	TP53	R148S	17	7577098	T	A	TP53	R148S	17	7577098	T	A	TP53	R148S
SW480	5	112175303	C	T	APC	Q1320*							5	112175303	C	T	APC	Q1320X
SW480	11	108201015	G	C	ATM	R2461P							11	108201015	G	C	ATM	R2461P
SW480	12	25398284	C	A	KRAS	G12D	12	25398284	C	A	KRAS	G12V	12	25398284	C	A	KRAS	G12V
SW480	17	7577120	C	T	TP53	R141H	17	7577120	C	T	TP53	R141H	17	7577120	C	T	TP53	R141H
